# Improvement of hand hygiene compliance in a private hospital using the Plan-Do-Check-Act (PDCA) method

**DOI:** 10.12669/pjms.35.3.6

**Published:** 2019

**Authors:** Aslihan Demirel

**Affiliations:** 1 *Aslihan Demirel, Department of Infectious Diseases, Istanbul Kadikoy Florence Nightingale Hospital, Istanbul, Turkey*

**Keywords:** Hand hygiene compliance, Hospital infection control, Plan-do-check-act (PDCA), Quality in health

## Abstract

**Objective:**

Improving the compliance to hand hygiene in healthcare providers is important to reduce healthcare-associated infections. This study aimed to compare the compliance rate before and after the improvement of compliance to hand hygiene.

**Methods:**

In this study 270 of the 348 medical staff working in a 61-bed private hospital was observed. The informed observation was performed by the infection control committee in the entire hospital using “Five Moments for Hand Hygiene” for a period of one year. After the first six months, an improvement study was conducted together with the hospital’s quality department using the plan-do-check-act cycle. The study was conducted in a private hospital in Istanbul/Turkey; Kadıkoy Florence Nightingale Hospital in 2014.

**Results:**

In the first six months of the year, 153 actions were observed at 316 proper situations. The compliance rate was 35%, 54% and 64% for the physicians, nurses and, other healthcare staff, respectively. The overall compliance rate was 48%. One hundred eighty-three actions were observed for 306 situations after the improvement and education studies. The compliance rate was 29%, 72% and 86%. The overall mean compliance rate was 60%.

**Conclusion:**

The promotion of hand hygiene requires the cooperation of the hospital administrators, infection control committee, and quality departments for better hand hygiene practices among the healthcare providers.

## INTRODUCTION

Healthcare-associated infections are one of the most important health quality indicators and lead to serious morbidity and mortality. Hand hygiene is the most effective, simplest, cheapest, but least compliant medical practice in preventing and controlling infections associated with health care.^1^,^2^ Healthcare staff, may be colonized with microorganisms identified as causative agents in hospital infections as a result of contact with patients and patient surroundings.^3^,^4^ Healthcare-associated infections are preventable complications in inpatients, and preventive measures are recommended as one of the most effective strategies to prevent infections.^5^ Many outbreaks in hospitals are usually associated with poor levels of compliance with hand hygiene.^4^,^6^ The most important approach to improve hand hygiene is surveillance of compliance, which is most often carried out by direct observation.^7^ The hospital infection control committee (HICC) and the quality department should analyze the reasons of poor compliance with hand hygiene and carry out solution-oriented approaches to improve hand hygiene compliance by means of collaboration with related department.^8^,^9^A limited number of data is available in our country, according to which the rate of infection is associated with health care.^10^ This study aimed to compare the compliance rate before and after the improvement of compliance to hand hygiene.

## METHODS

**Fig.1:**
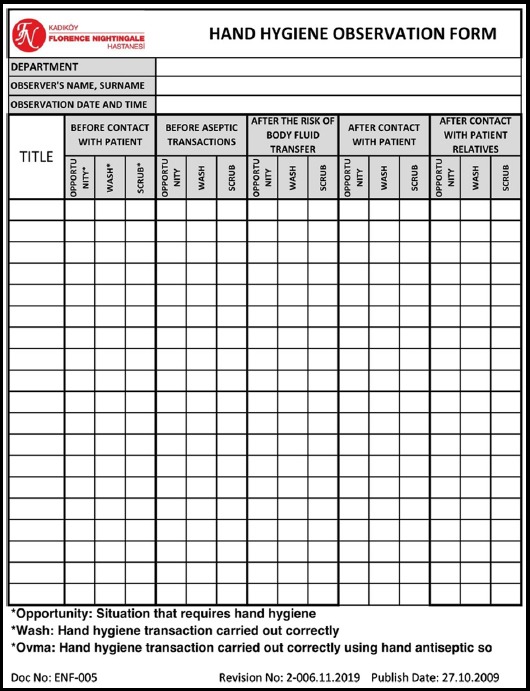
Hand hygiene observation form.

**Fig.2:**
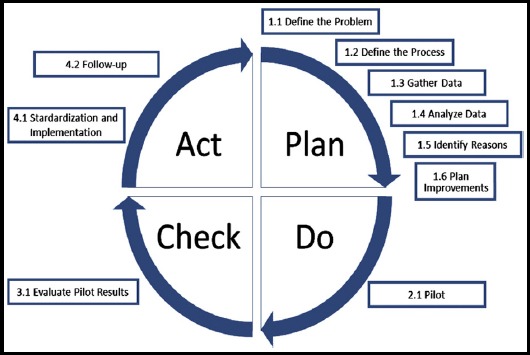
PDCA cycle.

In this study, 270 of 348 medical staff, working in a private hospital with 61 beds were observed. A total of 147 nurses, 72 physicians, 51 health care staff participated in the study. The informed observation was performed in the entire hospital using “Five Moments for Hand Hygiene” developed by the World Health Organization (WHO) for a period of one year.^8^ Five main rules defined by this strategy were to apply hand hygiene practice; once before patient contact, second before an aseptic task, third after patient contact, fourth after body fluid exposure risk, fifth after contact with patient surroundings. Hand hygiene observation form was prepared according to these rules. Overt observations were performed by the nurse in charge of wards under the leadership of HICC nurse. One nurse in charge of each ward has observed five to seven health care workers daily. The observation period took one-month to complete the observation of all the participants. Physicians, nurses and health care staff, including those working in outpatient clinics, were monitored for compliance with hand hygiene. Washing hands with water and soap or rubbing hands with alcohol-based antiseptics were acceptable methods of hand hygiene. Ten random samples from hands were obtained for bacterial cultures to observe the effectiveness of hand washing in each group of staff (compliant versus non-compliant). In order to reduce the turnover rate, nursing services and human resources department collaborated in conducting surveys on off times; interviews were arranged with those going off duty. Detailed inspections during this period revealed that the hand hygiene was known by the majority of health care personnel as only washing hands with water and soap, hand hygiene was mostly ignored before touching patient and during passing from patient to patient, hand disinfectant bottles were not suitable for use and hand hygiene was not applied after using gloves. The compliance rates of all personnel were recorded in the hand hygiene observation form (Fig.1). Hand hygiene compliance rates were recorded in the first six months afterwards; an improvement work was conducted together with our hospital quality department using the plan-do-check-act (PDCA) cycle (Fig.2). By using this method, the reasons for poor compliance were determined in the light of collected data; improvements were planned and started to be implemented. Hand hygiene compliance rates were observed for six months after the study. The EQUATOR checklist (Squire Checklist) was used to report this quality improvement work accurately.

## RESULTS

In the first six months of the year, 153 actions were observed at 316 appropriate moments. These observations included 39 physicians (28.2%), 82 nurses (58.9%) and 18 health care staff (12%). The compliance rate was 35% for the physicians, 54% for the nurses and 64% for the other assistant personnel. Mean compliance rate was 48% in all categories. As this rate was below 50%, it was decided to carry out some intervention in collaboration with the quality department. According to the PDCA cycle, the reasons of poor compliance were determined, which were high rates of personnel turnover (22%), poor awareness of the health care personnel on hand hygiene, fewer number of cautions reminders of hand hygiene, dissatisfaction of the health care personnel with soap and hand disinfectants and inadequate number of hand disinfectants in outpatient clinics. It was determined that the number of staff was 348 persons in the first six months of the year and 67 (19%) persons have received training on hand hygiene. After the interventions, the number of personnel working was 310 in the second 6 months of the year, and 231 (74.5%) persons were trained on hand hygiene. In the second half of the year, the turnover rate was reduced from 22% to 10% (Fig.2). As an improvement works the number of alcohol-based hand disinfectants was increased inwards and outpatient clinics.

**Fig.3:**
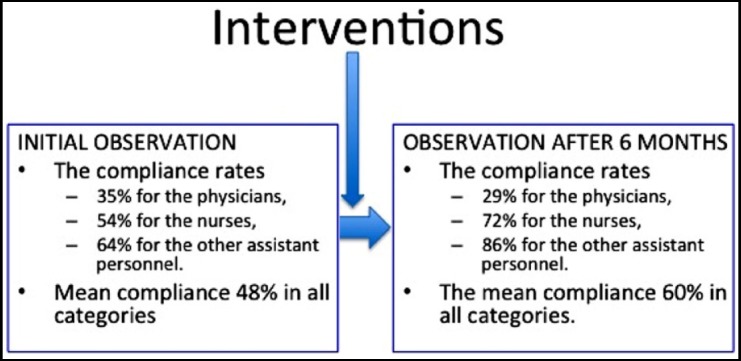
Initial observation, interventions and increasing compliance in second observation six months after.

Cautions such as posters, stickers were prepared to remind hand hygiene to health care personnel. The contents of the hand disinfectants and liquid soaps were re-evaluated and investigated in terms of causing skin irritation, and the companies were interviewed. Hand hygiene videos were displayed via intranet and hand wash instructions were revised. In the second half of the year after 183 actions were observed at 306 appropriate moments. These observations included 33 physicians (25%), 65 nurses (50%) and 33 health care staff (25%). The compliance rate of the physicians was 29%, the rate of nurses was 72%, and the rate of other assistant personnel was 86%. The mean compliance rate was found to be 60% in all categories (Fig.3).

## DISCUSSION

Infections associated with health care are the most important problems affecting the quality of health. Microorganisms are transmitted from one patient to another by the hands of health care personnel, leading to the emergence and spread of infections associated with health care.^9^ According to the European Center for Disease Control and Prevention report, 7% (3-10%) of patients in acute care hospitals in Europe develop infections associated with health care. A limited number of data is available in our country, according to which the rate of infection associated with health care has been reported as 13.4%.^10^ This rate increases to 48.7% in intensive care units.^11^ Hand hygiene has an important role in preventing infections associated with health care. All relevant national and international infection control organizations acknowledge the importance of hand hygiene in the prevention of infections. Despite the evidence demonstrating the importance of hand hygiene in preventing infections associated with health care, studies in the literature have shown that hand hygiene compliance of health care personnel was low.^1^,^2^,^6^,^12^ Studies conducted in our country have shown that the hand hygiene compliance rate of health care personnel was between 12% and 34%.^13^-^16^ Before compliance improvement interventions, the hand hygiene compliance rate was 48% in our center which is a private hospital. Various campaigns have been organized in order to increase hand hygiene around the world, and some studies have been conducted.^17^-^20^ In 2015, Hebert aimed to increase the hand hygiene compliance rate from 35% to over 80%, and after creating a team for this purpose, training, slogans, logos, posters, visual signs and videos have been prepared. With direct observation and the presence of role model, the hand hygiene compliance rate has been found to be 95.5% at the end of one year.^17^ Doron et al. have performed a versatile campaign based on training, observation and feedback and done marketing especially by using financial resources and working with advertising companies. As a result of this improvement work, the hand hygiene compliance rate was increased from 72% to 94% within six months.^18^ In studies conducted for increasing the compliance, alcohol-based hand antiseptic dispensers were spotted by placing flashing lights, and compliance with hand hygiene was significantly increased by such a simple and inexpensive method.^19^,^20^

In 2009, the ministry of health initiated campaigns with slogans and brochures to increase awareness of hand hygiene in our country. The awareness has raised through slogans such as “The danger is in your hands” and “Touch water and soap” and the regulations, such as providing training in hospitals on this subject for the quality in health, monitoring hand hygiene rates and sharing these rates with the ministry, have been established.

In some studies conducted to increase hand hygiene compliance, the PDCA cycle has been used.^21^,^22^ In a study conducted by Awaji et al., the roles of health care personnel in improving the compliance have been investigated, and the hand hygiene compliance rate has been increased about 15% within 10 days by patient training, patients’ warning of health care personnel, preparation of Arabic and English posters for patients.^21^ In an observational study conducted by Chen et al. in a stomatology hospital in China, the PDCA cycle has been used, and close observation has been made, the number of disinfectants has been increased, public meetings have been organized, and the compliance rate has been increased from 60% to 90%.^22^

In our hospital, a plan was made with the quality department to increase the hand hygiene compliance rate, and the PDCA cycle has been used in this work. However, further efforts should be made with observations, training and raising awareness in order to increase the rate of compliance.

After improvement works adherence to hand hygiene increased generally in healthcare workers but decreased from 35% to 29% in physicians. It has been suggested that this decrease in the rate of adherence in the physicians may be due to new physicians beginning to work in the hospital and incomplete education on hand hygiene of this new group.

## CONCLUSIONS

Increasing compliance with hand hygiene requires a long-term effort. Quality control works are important tools for health care personnel to understand and promote the importance of hand hygiene. Investigation of the reason for the poor compliance and versatile and systemic approaches would be more effective in increasing compliance. Although education is the simplest approach, easily accessible and available hand disinfectants and increasing awareness through various methods are important in increasing and maintaining compliance with hand hygiene.
